# Through the layers: how macrophages drive atherosclerosis across the vessel wall

**DOI:** 10.1172/JCI157011

**Published:** 2022-05-02

**Authors:** Leah I. Susser, Katey J. Rayner

**Affiliations:** 1 Department of Biochemistry, Microbiology and Immunology, Faculty of Medicine, University of Ottawa, Ottawa, Ontario, Canada.; 2 University of Ottawa Heart Institute, Ottawa, Ontario, Canada.; 3 Centre for Infection, Immunity and Inflammation, Faculty of Medicine, University of Ottawa, Ottawa, Ontario, Canada.

## Abstract

Cardiovascular disease (CVD) accounts for almost half of all deaths related to non-communicable disease worldwide, making it the single largest global cause of mortality. Although the risk factors for coronary artery disease — the most common cause of CVD — are well known and include hypertension, high cholesterol, age, and genetics, CVDs are now recognized as chronic inflammatory conditions. Arterial blockages, known as atherosclerosis, develop due to excess cholesterol accumulating within the arterial wall, creating a perpetually inflammatory state. The normally quiescent intimal layer of the vessel wall becomes laden with inflammatory cells, which alters the surrounding endothelial, smooth muscle, and extracellular matrix components to propagate disease. Macrophages, which can be either tissue resident or monocyte derived, are a key player in atherosclerotic disease progression and regression, and the understanding of their functions and origins continues to evolve with the use of deep phenotyping methodologies. This Review outlines how macrophages interact with each layer of the developing atherosclerotic plaque and discusses new concepts that are challenging our previous views on how macrophages function and our evolving understanding of the contribution of macrophages to disease.

## Macrophages and their classification 

Since their initial description in the late 19th century, the role of macrophages in inflammation and disease has been studied in depth; however, owing to their plasticity and tissue-specific functions, much remains to be defined. Macrophages are unique in that they are found in virtually all tissues and carry out diverse physiological roles integral to tissue homeostasis: phagocytosis of cellular debris and microbes, tissue repair, and the mounting of immune responses through cytokine production (1). With advancement in lineage tracing techniques in recent years, it is now understood that there are two major populations of macrophages: tissue-resident macrophages (TRMs), which are seeded early in fetal development, and monocyte-derived macrophages (MDMs), which, as the name suggests, are derived from the differentiation of monocytes when they exit the circulation into tissue. Both TRMs and MDMs act similarly as phagocytic cells that can mount an immune response while simultaneously promoting repair and homeostasis. However, TRMs have the capacity for self-renewal and take on specific roles and characteristics depending on tissue type. TRMs can be identified via shared expression signatures independent of tissue type in mice (e.g., CD64 and MerTK) but can be further marked by tissue-specific gene expression signatures (e.g., F4/80^+^Clec4F^+^ Kupffer cells in the liver; TRAP^+^CD61^+^ osteoclasts in bone) (2–5).

In contrast to TRMs seeded during development, MDMs are primarily derived from hematopoietic stem cells in the bone marrow and spleen, which differentiate into monocytes, then macrophages, in the presence of specific signals. In steady state, MDMs exist in low levels within tissues and are replenished through monocyte recruitment and differentiation via growth factors like macrophage colony-stimulating factor (6, 7). A subset of classical monocytes is ready to be recruited into the subendothelial space upon the first sign of inflammation or injury. These monocytes are marked by high expression of Ly6C (in mice) or CD14 (in human) and, depending on the stimulation they receive, can take on different phenotypes toward either proinflammatory (also known as M1/classically activated) or pro-resolving (also known as M2/alternatively activated). The extremes of these phenotypes have been extensively studied in cell culture, but MDMs will likely take on more transient versions of these phenotypes in vivo due to the complexity of stimulation they receive (8). In vitro M1 macrophages are polarized through the engagement of Toll-like receptor 4 (TLR4) by pathogen-associated molecular patterns, such as lipopolysaccharide derived from infection with Gram-negative bacteria or other receptors, e.g., by stimulation with IFN-γ. Upon polarization, M1 macrophages have increased bactericidal capacity, increased reactive oxygen species (ROS), a preference for glycolytic metabolism, release of numerous inflammatory cytokines, such as TNF-α and IL-1β, and expression of inducible nitric oxide synthase (iNOS) (9–11). Alternatively activated M2 macrophages become polarized upon IL-4 or IL-13 stimulation, promoting increased phagocytic capacity, a preference for oxidative metabolism, and a release of pro-resolving factors that promote angiogenesis, tissue repair, and immunoregulation, such as IL-10 and TGF-β (7). 

**Figure 1:**
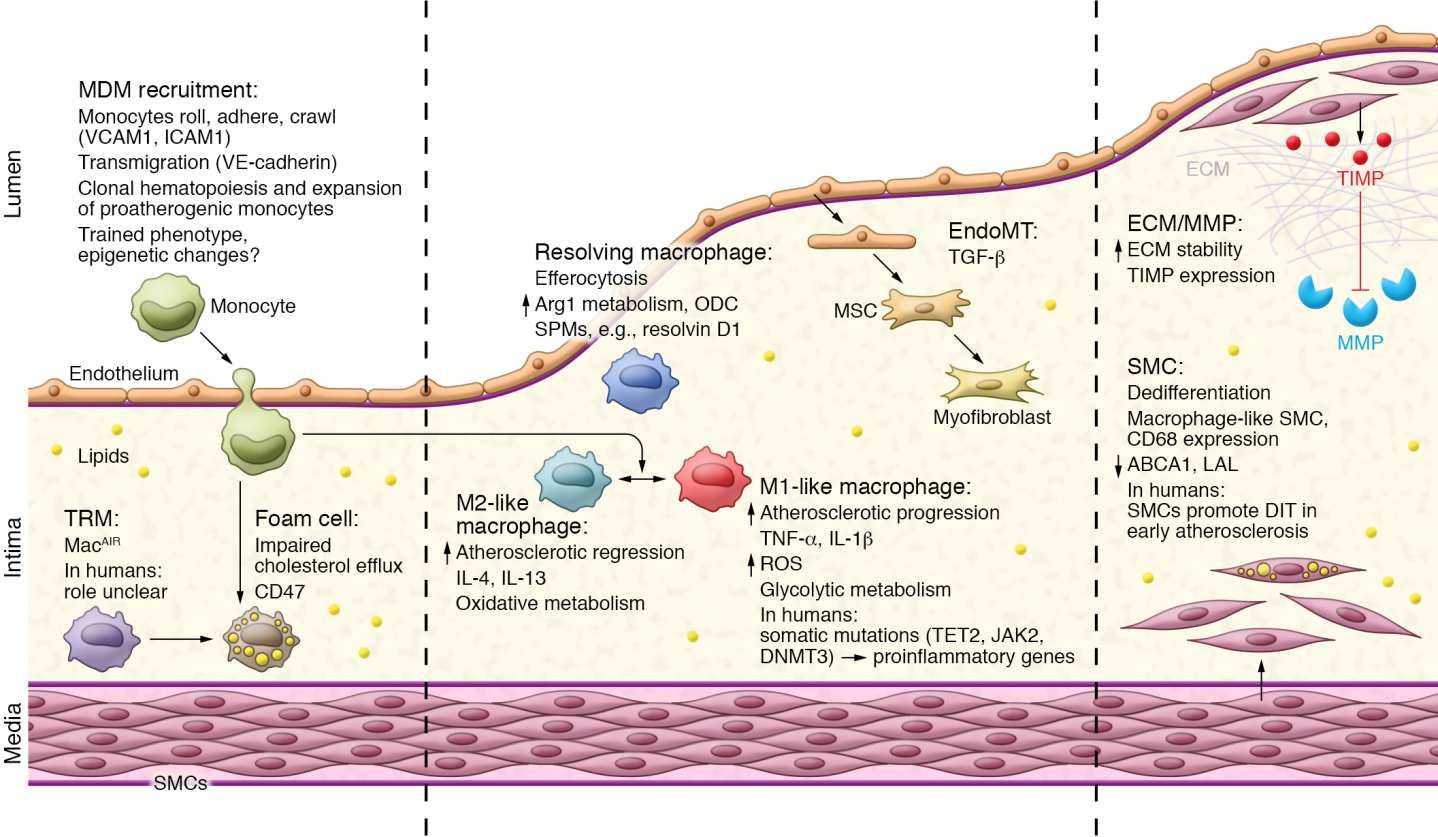
Overview of macrophage function through the stages of atherosclerosis. In the early stages of atherosclerosis, when cholesterol is abundant in the intima, MDMs are recruited via endothelial interactions and differentiation and, together with TRMs, engulf excess lipids to become foam cells. Certain macrophages adopt a proinflammatory M1-like phenotype that promotes inflammation and the formation of a necrotic core. During disease progression, endothelial cells can undergo EndoMT, and SMCs dedifferentiate into macrophage-like SMCs to become foam cells, all of which contribute to the growing plaque. To accommodate the growth in plaque size, ECM remodeling occurs through MMPs and, if the ECM is reduced and the SMC fibrous cap thins, plaques are prone to rupture. During disease regression and if cholesterol metabolism and efflux are efficient, macrophages take on a pro-resolving M2-like phenotype. An increase in M2-like macrophages alongside SPMs promotes inflammation resolution and plaque regression.

Our understanding of the unique properties of TRMs and MDMs has enabled a better understanding of macrophages in all diseases, including atherosclerosis (Figure 1). Proinflammatory or M1-like macrophages are commonly found in progressing plaques with active inflammation, and the reparative M2-like macrophages are seen more in regressing and stable plaques during repair (12–14). Macrophages may be the source of inflammation within a plaque, but they also change and shift phenotype according to the needs of the tissue and are required for the stabilization and resolution programs that accompany plaque regression in mice. Macrophages have complex and intricate dynamics within all the different components of an atherosclerotic plaque. This Review will summarize what is known about these complex dynamics layer by layer and discuss how this information may lead to the identification of novel targets that promote disease regression.

## Macrophages and their roles in atherosclerosis

### The endothelial-macrophage interface.

The endothelial monolayer lining the vascular lumen is the primary physical barrier between blood and tissue. The healthy endothelium regulates vascular tone and cell adhesion and maintains vascular homeostasis through anticoagulant, antithrombotic, and antiinflammatory activity. During atherosclerosis, these homeostatic properties are lost. Across the resting, nonstimulated endothelium, less than 1% of all patrolling monocytes are thought to cross into the subendothelial space (15). However, upon activation, like that which occurs in hypercholesterolemia or at sites of disturbed blood flow, monocytes are recruited. This recruitment of MDMs is a highly regulated process whereby monocytes roll, adhere, and crawl via interactions with endothelial cells (ECs), then transmigrate from the vascular lumen into the intima.

The release of inflammatory cytokines by the TRMs, such as TNF-α and IL-1β, induces the rapid expression of EC adhesion molecules like E- and P-selectin, intercellular adhesion molecule 1 (ICAM1), and vascular cell adhesion molecule 1 (VCAM1). This expression of adhesion molecules and the presentation of tethering chemokines by monocytes (i.e., CCL2, CCL5) allow them to roll on the vascular EC monolayer, overcoming the force of blood flow. Rolling is dependent on the interaction between selectins and monocyte P-selectin glycoprotein ligand 1 (PSGL1), VCAM1, monocyte-expressed very late antigen 4 (VLA4), and CD44, which enables monocytes to shift from a roll to firm attachment (16–19). C-C and C-X-C chemokines such as CCL2 and IL-8 also aid in monocyte firm attachment (20). Rolling and attachment are followed by crawling, a chemotactic step by which monocytes spread laterally to find the best location for transmigration. Intraluminal crawling depends on the monocyte factors lymphocyte function–associated antigen 1 (LFA1) and macrophage-1 antigen (Mac1) interacting with endothelial ICAM1 and ICAM2 (21). The final step of monocyte transmigration can happen at either transcellular or paracellular endothelial junctions. ECs lack proper tight junctions; instead, they have vascular endothelial cadherin (VE-cadherin), the gatekeeper of transmigration. Cell signals received from adherent monocytes disassociate adherens junctions through phosphorylation of VE-cadherin, allowing the macrophages to enter the subendothelial intimal space (22).

The diverse mechanisms that activate the expression of adhesion and transmigratory signals on ECs during atherosclerosis have been covered elsewhere (23) but begin when lipoproteins that become trapped in the subendothelial space are engulfed by TRMs, leading to the secretion of TNF-α and chemokines that act on luminal ECs to induce chemokines and adhesion molecules. Additionally, given that atherosclerosis predominantly forms at arterial branches and sites of disturbed/nonlaminar flow, the low–shear stress environment allows the expression of adhesion molecules and induces EC permeability via the developmental regulators TGF-β, BMP, and Wnt, among others (24). This notion is further supported by recent evidence that neuronal guidance cues (e.g., semaphorins, netrins, and ephrins), which normally act as migratory signals to neurons during development, play a prominent role in the interaction of ECs with monocytes in the atherosclerotic environment (25–28). Likely, the interplay between lipoprotein retention and low shear stress over the course of decades acts in concert to exacerbate plaque development: as the lesion grows, blood flow patterns are disturbed, activating EC permeability programs and allowing more passage of excess lipids; simultaneously, as ECs become more activated by TRMs in response to excess cholesterol, they express additional adhesion molecules and further increase permeability and immune cell recruitment.

The endothelium also plays a key role in the progression or regression of atherosclerosis through the release of extracellular vesicles (EVs). EVs are membrane-bound vesicles released as a form of intracellular communication to regulate homeostasis through the transport of microRNAs, lipids, and proteins (29). Endothelial cell–derived exosomes have been shown to contain microRNA-10a (miR-10a) and downregulate NF-κB signaling in monocytes, dampening the overall inflammatory response (30). Intravenous injection of endothelial cell–derived microvesicles (EMVs) containing miR-143/145 prevented smooth muscle cell (SMC) phenotypic switching and reduced atherosclerosis lesion size in *Apoe^–/–^* mice (31). While EMVs can be protective, there is also an association between elevated levels of EMVs in the blood and clinical atherosclerosis, which may mean EMVs could serve as a biomarker of disease (32). There is an intriguing suggestion that the success of statin therapies may be due to decreasing EMVs in the blood in addition to cholesterol lowering (33, 34). Lastly, endothelial cell–derived apoptotic transfer of miR-126 and miR-222 to other ECs has been shown to enhance EC proliferation and migration while inhibiting monocyte adhesion from controlling vascular homeostasis (35, 36). The study of EVs and how they impact atherosclerosis progression is continuously evolving, but it is clear that the contents of these vesicles dictate their roles. As the technology to better isolate and identify subpopulations of these vesicles develops, in addition to standardization of isolation protocols, so too will our understanding of their function and clinical utility.

Recently it was revealed that ECs contribute to the content of the plaque through endothelial-mesenchymal transition (EndoMT). EndoMT is the process by which ECs transition into multipotent mesenchymal stem cells, which can then differentiate into fibroblasts, SMCs, and osteogenic progenitors (37). In the context of atherosclerosis, loss of fibroblast growth factor receptor (FGFR) in ECs, which occurs in response to the inflammatory cytokines IL-1β, TNF-α, and IFN-γ found in the atherosclerotic plaque, activates TGF-β signaling, leading to a loss of endothelial phenotype and gain of mesenchymal phenotype. TGF-β promotes the activation of Smad-dependent and -independent pathways, inducing the expression of the transcription regulators Snail Snai1), Slug (Snai2), Twist, and Zeb, and resulting in activation of α-smooth muscle actin (αSMA, encoded by *ACTA2*) expression and loss of endothelial NOS (38, 39). FGFR expression can also be decreased by disruptions in laminar shear stress, as observed in atherosclerosis-prone areas of the arteries (40). This increase in EndoMT under chronic atherosclerosis conditions can further explain the fibroblasts found in unstable plaques, and the process of EndoMT alters the collagen/matrix metalloproteinase ratio, making the plaque more prone to rupture. On the other hand, EndoMT differentiation into SMCs is thought to promote plaque stability through the formation of a thick fibrous cap (41). TGF-β–mediated EndoMT also increases the expression of the monocyte adhesion molecules ICAM1 and VCAM1, promoting atherosclerosis progression through monocyte recruitment as outlined above (40).

### Macrophages in the intimal and medial layers.

The endothelium may be the primary barrier between the blood and the tissue, but the intima is where plaque fate and development ultimately occur. The retention of LDL-derived lipids from the circulation within the intima is the primary step in atherosclerotic lesion initiation. Under homeostatic conditions, lipids in the extracellular space are handled by TRMs via intracellular metabolic pathways (such as autophagy), storage (via lipid droplet formation), or efflux (via free cholesterol transfer to available HDL particles). During atherosclerosis progression, however, there is a lack of cholesterol management. The trapped LDL easily becomes modified by aggregation (agLDL) and/or oxidation (oxLDL) and thus becomes readily recognized by innate immune scavenger cells and TLRs. Additionally, large aggregates of LDL are digested by TRMs through the exocytosis of lysosomes, leading to cholesterol crystal formation and downstream activation of the inflammasome (42). TRMs attempt to efflux the excess lipid, but this process quickly becomes overwhelmed, transforming the macrophages into “foam cells” (43).

Efflux of cholesterol to HDL and/or its apolipoprotein A1 (apoA1) relies on the activity of the ATP-binding cassette transporters ABCG1 and ABCA1, respectively (44). When efflux acceptors are not available (e.g., low or dysfunctional HDL) (45, 46) or when the metabolism of lipid droplets is compromised (e.g., reduced autophagy) (47), ER stress is triggered and/or foam cells undergo programmed apoptotic (blebbing) or necroptotic cell death (rupture). Initially, apoptosis coupled with efficient efferocytosis by other macrophages keeps the plaque from growing. In late stages of atherosclerosis, when apoptosis is either overwhelmed or inhibited, foam cells can undergo necroptosis through the receptor-interacting serine/threonine-protein kinase 1 (RIPK1), RIPK3, and mixed-lineage kinase domain–like pseudokinase (MLKL) cascade, resulting in the bursting of the cellular membrane and release of its inflammatory content (48, 49). The released contents then act as an inflammatory signal for the recruitment of MDMs. Moreover, foam cells have high levels of CD47 (a potent “don’t eat me” signal) and reduced expression of MerTK (a key efferocytic receptor), rendering efferocytosis inefficient (50). In addition, other forms of cell death can contribute to lesion development. Pyroptosis is a form of inflammatory cell death mediated by caspase-1 whereby a pore-induced intracellular trap is created, releasing the cell’s cytosolic contents (51, 52). Ferroptosis, a more recently described form of inflammatory cell death induced by iron-dependent lipid peroxidation, has recently been proven critical in the initiation of atherosclerosis (53–55). The combined inefficiency in both efferocytosis and increased cell death, particularly necrotic forms of cell death, results in the formation of a necrotic core, a pocket of dead and dying macrophages, cell debris, and modified lipid (56, 57). The presence of a necrotic core is an indicator of an advanced and unstable lesion.

SMCs form the medial layer of healthy arteries and exist in a fully differentiated yet quiescent state, with contractile function marked by high levels of αSMA and smooth muscle myosin heavy chain (SM-MHC, encoded by *MYH11*) (58). During the early stages of atherosclerosis, medial SMCs dedifferentiate and migrate into the intimal space in the process of diffuse intimal thickening (DIT) (59–62). The early process of DIT is unique to human plaques, but intimal thickening can be experimentally induced in animals (62, 63). Intimal SMCs lose their contractile function, partly as a result of reduced expression of αSMA and SM-MHC, and acquire phenotypes reminiscent of fibroblasts, osteoblasts, and macrophages (64). A number of years ago, studies in vitro (65–67) and in mice (63, 68, 69) found that in proatherogenic environments, SMCs downregulate the contractile markers *Sm22a* (encoding SM22α, or transgelin) and *Acta2*, *Cd68*, and *Cd11b* (64). Loss of these key functional differentiation markers of SMCs occurs, at least in part, via epigenetic silencing of contractile gene promoters and activation of genes associated with stemness, resulting in dedifferentiated SMCs (70, 71). New evidence suggests that transitional SMCs in both mice and humans also have perturbed signaling in the retinoic acid pathway, which may be manipulated therapeutically to reduce atherosclerosis (72). Collectively, these findings have led to the realization that foam cells in the plaque that have historically been ascribed to TRM or MDM origin are, at least in part, transdifferentiated SMCs (73, 74). In humans, it is suggested that over 50% of foam cells in intermediate human coronary artery atherosclerosis are SMC-derived (74). This population of macrophage-like SMCs are more prone to become foam cells owing to lower expression of ABCA1 and lysosomal acid lipase (LAL), both integral for efficient cholesterol efflux (75). Moreover, SM22α is required for efficient nuclear translocation of LXRα, a key antiinflammatory transcription factor that promotes cholesterol efflux, further contributing to the foam cell phenotype of these cells (76). Transdifferentiated SMCs also have been shown to expand in a clonal manner akin to cancer (76–78). As with cancer, the clonal expansion provides several survival advantages, such as adaptations to toxic environments and suppression of efferocytosis by upregulation of CD47 (“don’t eat me” signal) (79).

Although macrophages in the intimal space have been the subject of intense study for almost a century, we are only now beginning to understand the diversity of macrophages and macrophage-like cells within the intima. Advances in the understanding of plaque development and its macrophage content have been possible because of the advancement in lineage tracing technology, which marks the cells in vivo along their differentiation pathway, making them easily identifiable by fluorescent or other genetic markers. This tracing technology, coupled with the power of single-cell RNA sequencing, has allowed for an unprecedented, detailed mapping of all the cells in a tissue or sample. Entire systems have been mapped through this technique, such as the hematopoietic system, lung, kidney, and heart (80). Through this, we have uncovered that the intima of the plaque is more complex than once believed. Notable examples include the realization that, as described in the section above, SMCs adopt a macrophage-like phenotype and express CD68 during the progression of atherosclerosis (64, 68, 70, 71, 73, 74). Fate mapping and high-resolution transcriptomics also allowed for the identification of aortic intima-resident macrophages, also known as Mac^AIR^ cells, a unique subtype of macrophages that are deposited in the aortic arch at birth and maintained through CSF1-dependent local proliferation and that are largely replaced by recruited MDMs during atherosclerosis progression (81). These Mac^AIR^ cells were originally categorized as the dendritic cells that were thought to be the earliest phagocytes in the aortic arch in mice. The contribution of local proliferation versus MDM replacement from the circulation in maintaining this distinct cell cluster is still under debate (81). What was surprising in this study was that Mac^AIR^ cells were found to express high levels of *Il1b* transcript, but almost undetectable *Nlrp3*, a component of the inflammasome complex necessary for IL-1β activation. Therefore, unlike what was previously assumed about macrophages in progressing mouse atherosclerotic plaques, there are distinct subtypes that preferentially take up excess lipid or that produce potent proinflammatory cytokines. Many of these observations had been made previously using other techniques, but the combination of lineage tracing and high-resolution transcriptomics has allowed for a more refined characterization of these cells (80, 81, 82).

### Extracellular matrix and macrophage interactions.

Another macrophage relationship that has a direct role in atherosclerosis progression is the interaction of macrophages with the extracellular matrix (ECM), composed of biologically active proteins like collagens (I, III, IV, and V), elastin, glycoproteins (fibronectin, thrombospondin, vitronectin, and osteopontin), and proteoglycans, which confers tensile strength and elasticity to the arterial wall (83). During the early stages of atherosclerosis, the ECM acts as a net, trapping cholesterol within the arterial wall. The proteoglycans in the subendothelial space interact directly with apoB on the LDL particle because of its strong positive charge and the negative charge of proteoglycans — the so-called response-to-retention hypothesis (84). ECM components in the subendothelial space are primarily secreted by modulated SMCs, while matrix metalloproteinases (MMPs) — enzymes that cleave ECM and other proteins — are released primarily by macrophages. The ratio of ECM to MMP is tightly regulated and is sensitive to inflammatory stimulation such as TGF-β, promoting collagen and elastin synthesis. In addition, tissue inhibitors of metalloproteinases (TIMPs) are secreted by those same cells as endogenous inhibitors to allow for quick regulation of MMP-mediated ECM remodeling (83). TGF-β is produced by ECs or other cells; this enhances collagen production, while proinflammatory TNF-α and IFN-γ secreted by inflammatory cells decrease collagen synthesis (83, 85). In parallel, when MMPs are higher because of the presence of macrophages in the lesion, this is associated with decreased plaque stability through both a reduction in structure and the release of inflammatory cytokines, which also get trapped within the ECM. When there are fewer MMPs, the plaque is more stable through the ECM, helping form a robust fibrous cap (85). As such, the communication and interplay between macrophages and SMCs dictate the state of the ECM and thus the structural stability of a plaque.

### Interactions with other immune cells.

During atherosclerosis progression, macrophages interact with other cells of the innate and adaptive immune systems. These pathways and interactions have been covered extensively in other reviews (12, 86, 87), but there is an increasing appreciation for the adaptive immune system in the clinical contribution to and protection from atherosclerosis. By virtue of being antigen-presenting cells, macrophages and dendritic cells present oxLDL as a foreign body on their major histocompatibility complex to activate T lymphocytes, further stimulating B cell activation (88–90). M1 macrophages and Th1 cells promote atherosclerosis by producing proinflammatory cytokines and chemokines, while M2 macrophages, Tregs, and B-1 cells suppress inflammation, reduce plaque size, and promote plaque stability (91, 92). IgM autoantibodies, which can be endogenously produced and react to the oxidized phospholipid found on oxLDL, have recently been applied as a treatment for advanced stages of atherosclerosis (93–96). Cherepanova et al. generated a novel IgM autoantibody, 10C12, targeting similar oxidized phospholipids and showed that administration of this autoantibody in *Igm^–/–^ Apoe*^–/–^ mice fed a low-fat diet led to a 40% decrease in lipid accumulation within the aorta (95). Using a similar concept, Zhang et al. developed a novel human single-chain variable fragment antibody against an oxidation epitope of oxLDL called ASA6, which decreased atherosclerotic lesion area in *Apoe^–/–^* mice by regulating fatty acid metabolism and inhibiting M1 macrophage polarization (97). In addition, conjugation of ASA6 to MRI/near-infrared II (NIR-II) dual-function nanoparticles enabled a dual therapy and diagnostic tool, allowing for noninvasive imaging of atherosclerotic lesions — something that has been very challenging previously (97).

### Macrophages in the regression and resolution of atherosclerosis.

Although accumulation of macrophages and their interactions with other cells in the vessel wall directly cause atherosclerosis, they are also necessary for the stabilization and regression of plaques. The process of atherosclerosis regression requires newly recruited monocytes from the circulation in response to CCR2, and without these MDMs, regression does not occur (7). In contrast, MDMs unable to become inflammatory macrophages (e.g., by deletion of TRAM downstream of TLR4 activation or deletion of the proinflammatory neuronal guidance cue netrin-1) can be pushed toward the pro-resolving phenotype to promote regression of atherosclerosis (98, 99). Both clinically and experimentally, the main mechanism by which existing atherosclerotic lesions begin to regress and repair is through lowering of circulating plasma lipid. In mouse models, this is usually accompanied by the removal of excess lipids from foam cells by increasing cholesterol efflux via ABCA1 to apoA1/HDL via the reverse cholesterol transport pathway (100). When cholesterol efflux is activated in settings of high HDL, plaque macrophages take on a pro-resolving M2-like phenotype, releasing antiinflammatory cytokines such as IL-10 and TGF-β, thereby promoting tissue repair and angiogenesis (101). The pro-resolving phenotype also promotes phagocytosis of debris and efferocytosis of apoptotic cells, helping to reduce the necrotic core. Indeed, the efferocytosis and degradation of apoptotic cells promote macrophage proliferation, improving the number of macrophages available for efferocytosis, further promoting the regression process (102).

To mount an effective efferocytic response during inflammatory resolution, macrophages alter their metabolic profile to favor oxidative metabolism and activation of the phagolysosome to degrade engulfed apoptotic cells. Without full efferocytic capacity, or when the metabolic requirements for efferocytosis are not met, atherosclerosis regression cannot occur (103). During resolution of inflammation, macrophages produce polyamines like putrescine, spermidine, and spermine to support proliferation and other processes during tissue repair. In the atherosclerotic environment, pro-resolving macrophages require arginine — the precursor for polyamines — to continually efferocytose dead cells and regress atherosclerosis. Loss of the expression of arginase 1 (*Arg1*) and ornithine decarboxylase (*Odc*) in macrophages impairs atherosclerosis regression in mice, owing to defective MerTK expression and inefficient efferocytosis (104, 105). Conversely, changes in the metabolic milieu can also alter macrophage pro- and antiinflammatory responses during atherosclerosis progression. Desmosterol, a precursor in the cholesterol biosynthetic pathway, has been found to reduce ROS and NLRP3 inflammasome activation in mice, and blocking desmosterol signaling promotes atherosclerosis (106–108). Another product in the cholesterol biosynthetic pathway, mevalonate, can trigger epigenetic reprogramming and inflammatory activation of macrophages via trained immunity, and blocking mevalonate production with statins reduces this inflammatory activation (109). These studies collectively indicate that MDMs are likely flexible to adopt a pro- or anti-atherosclerotic phenotype, depending on the inflammatory signals received, the metabolic environment, and the expression of necessary machinery to mount a progression or regression response.

The contribution of non-macrophage foam cells, like transdifferentiated SMCs, to atherosclerosis regression has not been fully investigated; however, transdifferentiated SMC foam cells in both mice and humans express low levels of ABCA1 compared with macrophages, which suggests that approaches that promote cholesterol efflux to regress existing plaques would be defective in lesions rich in SMC foam cells (110). On the other hand, SMCs from both the medial and intimal layers contribute to plaque stability by forming a fibrous cap through the secretion of ECM proteins (64). Therefore, controlling the phenotype of SMCs within the lesion is essential for ensuring the removal of excess cholesterol and promoting plaque stability, and both of these processes are important therapeutic targets. And just like the crosstalk between macrophages and T cells that is necessary for the progression of inflammatory atherosclerosis, the interaction of T cells, specifically Tregs, with macrophages in regressing plaques is necessary to activate M2 macrophage resolution programs (111). Therefore, macrophages cannot be considered only as perpetrators of inflammation and disease in the vessel wall but instead are equally responsible for the reversal and stabilization of disease that are of utmost therapeutic importance.

Specialized pro-resolving mediators (SPMs) have recently emerged as a new therapeutic target to promote inflammation resolution and regression. SPMs are a subclass of lipid mediators derived from arachidonate, eicosapentaenoic acid, docosahexaenoic acid, or *n*-3 docosapentaenoic acid that are metabolized into lipoxins, resolvins, protectins, and maresins (112). Comparing stable versus unstable regions of human atherosclerotic lesions, there is an imbalance between pro-resolving SPMs and proinflammatory lipids; specifically, regions of plaque instability have a higher ratio of leukotriene B_4_ (LTB4) to resolvin D1 (RvD1). In *Ldlr^–/–^* mice with advanced lesions, administration of RvD1 restored the RvD1/LTB4 ratio, decreased the necrotic core, and increased fibrous cap formation, thus increasing plaque stability. Interestingly, this increase in fibrous cap thickness was not due to a change in either SMC or macrophage content overall; instead, it is believed that SPMs specifically promoted the resolution of inflammatory cells from the plaque and changed their phenotype to a pro-resolving program (113). SPMs act directly via their cell-surface receptors to promote macrophage phagocytic clearance and efferocytosis, which subsequently feed back to increase SPM biosynthesis, synergistically promoting atherosclerosis regression. In the context of advanced atherosclerosis, RvD1 can also promote the clearance of necroptotic cells by overcoming the CD47 “don’t eat me” signal through ER-mediated phagocytosis (50). New roles for SPMs continue to emerge as we begin to study this class of lipid mediators, and RvD1 in particular is becoming a very promising new therapeutic target.

## Emerging concepts about vascular macrophages

Our understanding of vascular macrophages and the role they play in atherosclerosis development has evolved greatly over the past decade. Until recently, macrophages were identified almost exclusively by their expression of surface markers, such as CD68. And while high-resolution sequencing has allowed for a more unbiased definition of cell types using the aggregate of their transcriptomic expression signatures rather than a historical, predefined marker, one major limitation is that this sequencing is often done at a single time point in disease progression, making it challenging to understand the kinetics of transitional cell phenotypes. As single-cell transcriptomics becomes more affordable and accessible and is combined with other high-resolution approaches (e.g., spatial transcriptomics, single-cell proteomics), we will undoubtedly learn even more about the functional consequences, if any, of these diverse cell types on atherosclerosis. Do different subtypes of macrophages interact differently with other immune cells? Do these subtypes have different capacities for cholesterol efflux or resolution, two critical components of the regression of atherosclerosis?

An ongoing challenge in understanding macrophage subtypes and phenotypes is the lack of data from human disease. Many of the in-depth studies described above were performed in mice, and correlations were made to humans; it is not clear whether the same factors that influence macrophage interactions in the vessel wall also occur in human disease. For example, the exact quantity and role of TRMs in the early phases of plaque development in humans are under debate, as the characteristics of mouse TRMs differ in human plaques (114). Recently, a newly identified risk factor known as clonal hematopoiesis of indeterminate potential (CHIP) (115) was found to be strongly associated with coronary heart disease in humans (116). When CHIP occurs, hematopoietic stem cells acquire somatic mutations that provide a survival advantage, resulting in clonal expansion of altered myeloid cells with enhanced proinflammatory and proliferative capacity (117). These clonal cells expand with age and increase the risk of cancer, cardiovascular disease, and death (116). The majority of mutations are found within the *TET2*, *DNMT3A*, and *JAK2* genes. Patients with *TET2* or *DNMT3A* mutations have poorer prognosis and long-term clinical outcomes for chronic heart failure (115). *Tet2* (heterozygous and homozygous) mutations accelerated and worsened atherosclerosis progression in mice (118). *Dnmt3a* deletion in macrophages was shown to promote inflammation (119), and *Jax2* gain-of-function mutation was shown to increase atherosclerotic lesion size in mice (120). Now, as we begin to unravel the complexities of macrophages in the vessel wall, it remains to be seen whether CHIP influences macrophage subtypes and their interactions with vascular cells. Given that CHIP is predominantly associated with age, it is a distinct possibility that the biology of MDMs versus TRMs in young versus aged individuals is differentially impacted by CHIP. Further functional analysis of each subtype in aged and young populations would greatly expand our understanding of atherosclerosis moving forward.

Little is known about how external or environmental perturbations, such as exercise, diet, or infections, may alter these newly identified macrophage subtypes in the vessel wall. Indeed, lifestyle factors influence the release of monocytes into the circulation and can either accelerate disease (e.g., perturbed sleep, psychological stress; refs. 121, 122) or reduce disease progression (e.g., exercise or healthy diets; refs. 123, 124). The long-term influence of the environment on disease progression has been demonstrated to alter the epigenetic landscape of immune cells. In mice, external challenges like long-term high-fat-diet feeding and chronic stress induce transcriptional reprogramming of myeloid progenitor cells due to changes in the histone methylation patterns. The reprogramming, or “memory” of the environmental challenge, results in increased monocyte proliferation and augmented innate immune cytokine production, promoting atherosclerosis development (125, 126). In humans, a similar phenotype is observed in bone marrow and circulating progenitor cells from patients with coronary disease, which showed enhanced cytokine production upon stimulation compared with cells from control patients (127). Once we have a better understanding of the phenotypic and functional landscape of newly identified macrophage subtypes and how they precisely propagate or protect from disease, we can determine whether modifiable risk factors directly influence disease by cell-specific mechanisms.

The gold standard for treating atherosclerosis are lipid-lowering therapies, such as statins, to target high circulating cholesterol and thus ultimately reduce the underlying cause of inflammation in the arteries. However, a significant disease burden remains in many individuals even when cholesterol is low; therefore, there is now a push toward developing therapies that directly target inflammation (128). Canakinumab, an antibody against IL-1β, has been shown to reduce recurrent cardiovascular events in the CANTOS (Canakinumab Anti-inflammatory Thrombosis Outcomes Study) trial. This trial proved the efficacy of targeting inflammation as a therapy or the treatment of cardiovascular disease (129). Colchicine, a widely available, safe, and low-cost antiinflammatory, was investigated as another potential therapy. The LoDoCo (Low-Dose Colchicine for Secondary Prevention of Cardiovascular Disease) trial showed a significant reduction in recurrent cardiovascular events over a 3-year follow-up when colchicine was added to statin therapy (128, 130). These two very successful trials have demonstrated proof of concept of the targeting of macrophages and the inflammasome pathway. However, there are limitations with broad-spectrum antiinflammatory drugs, including risk of serious infection and off-target effects. To overcome these challenges, one approach is to use targeted nanoparticles to directly deliver medicines to the atherosclerotic plaque. To this end, Tao et al. developed siRNA nanoparticles targeting CaMKIIγ in lesional macrophages, which impairs efferocytosis through the MerTK pathway, leading to fibrous cap thinning and an enlarged necrotic core, indicating a more vulnerable plaque prone to rupture. These siRNA nanoparticles were shown to reduce CaMKIIγ expression in mouse aortic lesions and promote plaque stability (131). This combination of macrophage-targeted nanoparticles containing inflammation-targeted therapies holds promise for reducing inflammation and improving lesion stability without weakening host defense.

## Conclusion

Vascular macrophages, derived either from monocytes in the circulation or at birth as tissue-resident cells, impact and direct atherosclerosis through their interaction and communication with all the cell types in the vessel wall. Through interaction with ECs, macrophages get recruited to the subendothelial space, promote EndoMT, and accumulate in the nascent plaque. Intimal macrophages control the progression or regression of disease through their intercellular communication, cholesterol management, and influence on the ratio of MMPs to ECM, affecting plaque stability. Lastly, macrophages communicate with other immune cells to direct atherosclerosis progression and regression and are critical for both processes. The diverse and dynamic relationship of vascular macrophages with other cells in the vessel wall continues to be elucidated, and with the advent of high-throughput and -resolution sequencing and imaging tools, we continually learn more about macrophages in the vascular environment. Together with a better understanding of the diverse phenotypic and functional properties of macrophages in human atherosclerosis, this will one day allow us to develop better macrophage-targeted therapies for vascular diseases.

## Version 1 05/02/2022

Electronic publication
